# Optimizing health-related quality of life assessments for stroke survivors: a validation study of psychometric properties for the Vietnamese version of stroke impact scale 3.0

**DOI:** 10.3389/fpubh.2025.1570980

**Published:** 2025-05-20

**Authors:** Thao Thi Phuong Nguyen, Hai Bui Hoang, Huyen Thi Thanh Vu, Seung Won Lee

**Affiliations:** ^1^Academy of Medical Sciences, Ho Chi Minh City, Vietnam; ^2^Institute for Preventive Medicine and Public Health, Hanoi Medical University, Hanoi, Vietnam; ^3^Department of Emergency and Critical Care Medicine, Hanoi Medical University, Hanoi, Vietnam; ^4^Department of Emergency and Critical Care, Hanoi Medical University Hospital, Hanoi Medical University, Hanoi, Vietnam; ^5^Department of Geriatrics, Hanoi Medical University, Hanoi, Vietnam; ^6^Department of Scientific Research, National Geriatric Hospital, Hanoi, Vietnam; ^7^Department of Precision Medicine, Sungkyunkwan University School of Medicine, Suwon, Republic of Korea; ^8^Department of Artificial Intelligence, Sungkyunkwan University, Suwon, Republic of Korea; ^9^Department of Metabiohealth, Sungkyunkwan University, Suwon, Republic of Korea; ^10^Personalized Cancer Immunotherapy Research Center, Sungkyunkwan University School of Medicine, Suwon, Republic of Korea

**Keywords:** stroke, health-related quality of life, validation studies as topic, psychometric, Vietnam

## Abstract

**Background:**

Ensuring lifelong health among aging populations necessitates comprehensive assessments of functional recovery and quality of life, particularly for vulnerable groups such as older adult stroke survivors. While the Stroke Impact Scale (SIS) 3.0 is a widely validated instrument for evaluating health-related quality of life (HRQoL) in stroke survivors, its psychometric properties have not yet been examined in the Vietnamese context. This study aimed to translate, culturally adapt, and validate the Vietnamese version of the SIS 3.0 (V-SIS 3.0), providing a robust tool to support holistic, multidimensional approaches to stroke rehabilitation in aging populations.

**Methods:**

A cross-sectional study was conducted from July to December 2021 at the National Geriatric Hospital in Hanoi, Vietnam. The study enrolled 256 stroke survivors aged 45 years or older who had experienced a stroke between 1 month and 1 year prior to participation. The V-SIS 3.0 questionnaire was developed through a rigorous forward and backward translation process. Its factorial structure was examined using exploratory factor analysis (EFA) and confirmatory factor analysis (CFA). Internal consistency was assessed via Cronbach’s alpha, and convergent and divergent validity were evaluated through correlation analyses. Additionally, Item Response Theory (IRT) was employed to examine item discrimination and difficulty.

**Results:**

EFA identified a four-factor structure consisting of Physical (28 items), Cognitive (12 items), Social Participation (10 items), and Emotional (8 items) domains. CFA supported this structure, indicating a good model fit (RMSEA = 0.080, CFI = 0.925, TLI = 0.918, SRMR = 0.053). The instrument demonstrated excellent internal consistency across all domains, with Cronbach’s alpha values of 0.971 for Physical, 0.950 for Cognitive, 0.949 for Social Participation, and 0.920 for Emotional. Convergent and divergent validity were confirmed by strong item correlations within each factor, while IRT analysis further indicated high discrimination and appropriate difficulty levels for most items.

**Conclusion:**

The V-SIS 3.0 is the first culturally adapted and validated tool to assess HRQoL in Vietnamese stroke survivors. By offering a reliable, multidimensional evaluation of physical, cognitive, emotional, and social wellbeing, this instrument enhances clinical assessments, informs targeted interventions, and ultimately contributes to more effective aging and lifestyle strategies for stroke survivors in Vietnam.

## 1 Introduction

As global life expectancy increases, promoting lifelong health and wellbeing has become a critical public health priority—especially for aging populations facing chronic conditions like stroke. In Vietnam, stroke is the leading cause of disability and mortality, with incidence and prevalence rates reported at 161 and 415 per 100,000 individuals, respectively (1, 2). In addition, infections acquired during hospitalization can significantly impede progress in rehabilitation and recovery (3). Many stroke survivors experience long-term challenges, including physical impairments, cognitive decline, emotional distress, and difficulties with social reintegration, all of which significantly compromise their health-related quality of life (HRQoL) (4). Addressing these multifaceted impacts requires an integrated approach that incorporates comprehensive assessment tools to guide effective rehabilitation and lifestyle interventions.

Historically, stroke research has concentrated on first-year mortality, recurrence, and disability, leading to the development of assessment tools that primarily measure neurological impairment and physical limitations (5, 6). However, recent trends have increasingly emphasized patient-centered outcomes, such as health-related quality of life, self-perception, and overall wellbeing. For example, a systematic review of Medline data up to March 2008 identified 1,940 articles containing the keywords “quality of life” and “stroke,” highlighting the shift toward a more holistic view of recovery that goes beyond traditional clinical measures (5).

To support a multidimensional approach to stroke recovery, the Stroke Impact Scale (SIS) was developed as a comprehensive tool to assess the wide-ranging effects of stroke on HRQoL. Developed by Duncan et al. (7) at the University of Kansas Medical Center, the SIS captures the physical, emotional, and social impacts experienced by stroke survivors (7). Previous studies have examined the reliability, validity, and psychometric properties of SIS 3.0 in various languages, including Nigerian (8), German (9), Korean (10), Moroccan (11), and Brazilian (12).

Despite its widespread use, the Vietnamese version of the Stroke Impact Scale 3.0 (V-SIS 3.0) has not yet undergone validation, limiting its applicability in Vietnam’s healthcare and rehabilitation settings. Cultural and linguistic nuances can significantly affect how individuals interpret health-related questions, and direct translations without rigorous validation risk yielding inaccurate assessments. Establishing the reliability and validity of the V-SIS 3.0 is therefore essential for effectively evaluating HRQoL among Vietnamese stroke survivors. This study aimed to develop the V-SIS 3.0 through a culturally sensitive translation and adaptation process and to assess its validity, reliability, and psychometric properties in a hospital setting. By providing a robust, standardized tool for assessing HRQoL, this research supports integrated strategies for lifelong health and enables more personalized rehabilitation and lifestyle interventions for stroke survivors in Vietnam.

## 2 Materials and methods

### 2.1 Study design and participants

Between July 1 and December 31, 2021, a cross-sectional study was conducted at the National Geriatric Hospital in Hanoi, Vietnam, focusing on stroke survivors. Eligible participants were 45 years or older and had experienced a stroke between 1 month and 1 year prior to the study. Individuals with unstable medical conditions, other neurological diseases, transient ischemic attacks, preexisting mood or cognitive disorders, or those taking anxiolytics, antidepressants, or antipsychotics—or those with consciousness disorders such as coma or severe cognitive impairment—were excluded. Of the 293 individuals invited to participate, 256 completed the questionnaire, resulting in a response rate of 87.37%.

### 2.2 Measurements and instruments

A comprehensive questionnaire was designed to collect information on demographics, stroke-related details, health conditions, and responses to the V-SIS 3.0. Five trained nurses conducted interviews, each lasting between 35 and 40 min. A pilot survey with 10 stroke patients was carried out to ensure clarity and appropriateness, leading to final revisions of the instrument. Data from the pilot study were excluded from the primary analysis, and full data collection commenced only after confirming that no technical issues were present.

#### 2.2.1 Outcome variables

*Stroke impact scale 3.0:* the SIS 3.0 is a self-report instrument comprising 59 items designed to assess stroke outcomes and measure HRQoL. It covers eight domains: strength (four items), hand function (five items), mobility (nine items), instrumental activities of daily living (10 items), memory and thinking (seven items), communication (seven items), emotion (nine items), and social participation (eight items). Each domain is scored on a scale from 0 to 100, with higher scores reflecting better HRQoL outcomes (13).

*Translation procedures of SIS 3.0:* the translation process was designed to ensure that the language is clear and culturally appropriate for the Vietnamese context (Supplementary Table 1). To translate the SIS 3.0 into Vietnamese, two native speakers proficient in English independently completed the translation. This process was overseen by two rehabilitation physicians and a psychologist, all under the supervision of the principal investigator, who reached a consensus on the initial Vietnamese version. A separate native Vietnamese speaker with excellent English skills and no prior exposure to the original SIS 3.0 then performed a back-translation into English. The back-translated version was compared to the original instrument, and any discrepancies were addressed through necessary revisions.

#### 2.2.2 Covariates

*Demographic variables:* participants provided demographic details, including age, sex (male or female), employment status (employed or unemployed), educational level (secondary school/lower secondary or high school/higher education), and marital status (living with a spouse or single, widowed/widower).

*Stroke-related characteristics:* information about stroke characteristics was gathered based on several factors: (i) etiology (ischemic/ hemorrhagic/ or unknown) as recorded in medical documents, (ii) number of stroke events (single occurrence/ multiple occurrences), (iii) time from stroke onset to study participation (< 1 month/ 1 month to < 3 months/ 3 months to < 6 months/ 6 months to 1 year); and (iv) hemiplegic side (right/left).

*Modified Rankin scale (MRS):* the disability of stroke survivors was evaluated using the Modified Rankin Scale (MRS), which ranges from no symptoms (0 points) to severe disability (5 points).

### 2.3 Statistical analysis

STATA version 15.1 (Stata Corp. LP, College Station, USA) was used for data analysis. The listwise deletion method was employed to handle missing data before analysis. Continuous variables were presented as means (standard deviations), while categorical variables were expressed as frequencies (percentages). Skewness and kurtosis coefficients were reported following their calculation. Floor and ceiling effects were identified when more than 15% of participants selected the lowest score [1] or highest score [5] response options (14). A *p*-value < 0.05 was considered statistically significant.

#### 2.3.1 Reliability

Cronbach’s alpha was used to assess internal consistency reliability, with values above 0.70 considered acceptable (15). Additionally, we examined correlations between domains, between individual items, and between each item and the total score. We also calculated Cronbach’s alpha for each domain with the exclusion of specific items to evaluate their individual contributions to the overall reliability.

#### 2.3.2 Factorial structure

**Figure 1:**
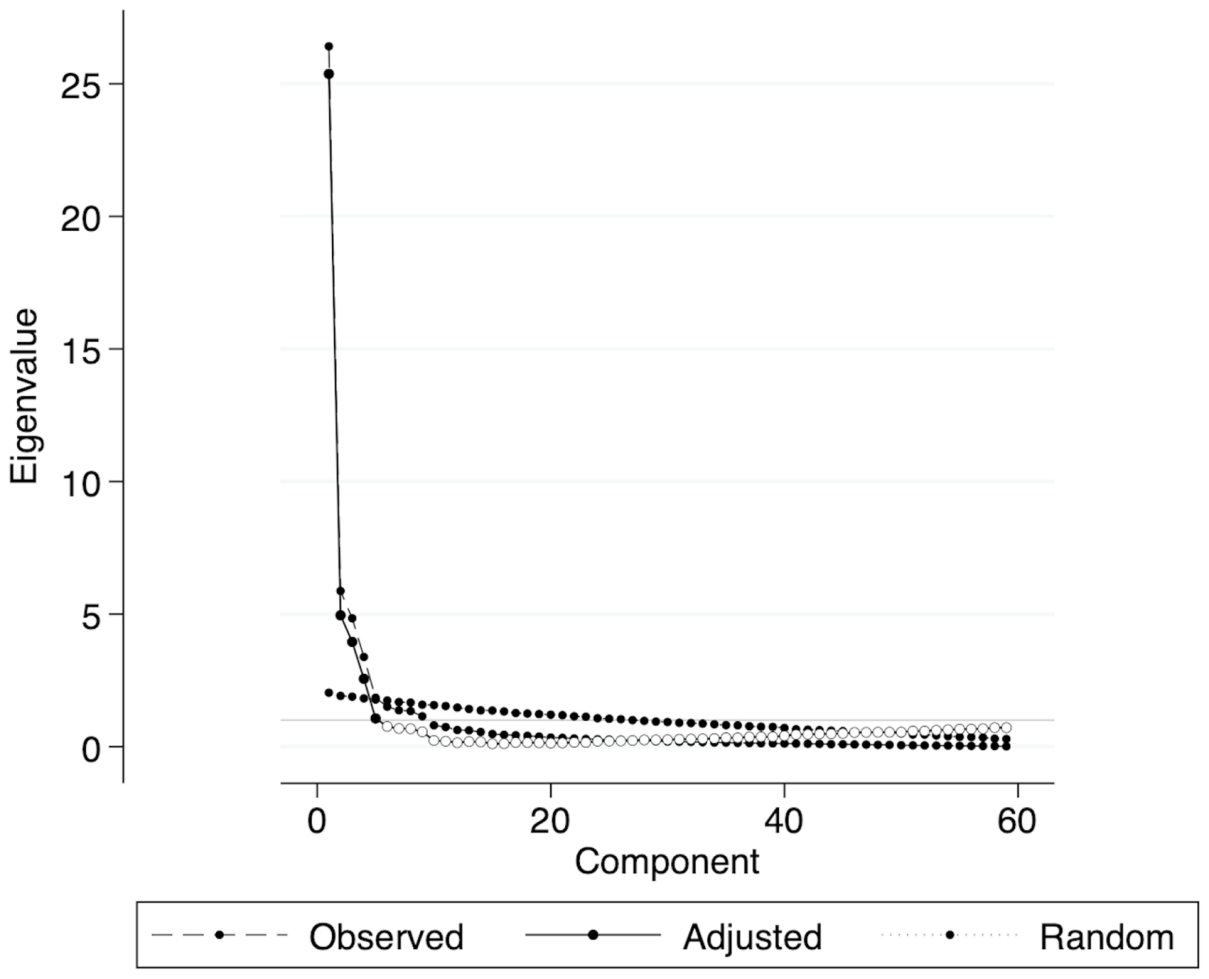
Scree parallel plot of 59 eigenvalues. The plot shows observed, adjusted, and random eigenvalues, aiding in determining meaningful components based on the parallel analysis criterion. Components above the random eigenvalue line are retained.

To identify the optimal structural model for the V-SIS 3.0, this study conducted exploratory factor analysis (EFA) using principal component analysis (PCA) on the observed data. The number of factors was determined using multiple methods: the Scree plot, parallel analysis (Figure 1), eigenvalues, and the proportion of variance explained. Specifically, we looked for the elbow point in the Scree plot, retained factors with eigenvalues greater than those from parallel analysis and the Kaiser-Guttman criterion, and considered the cumulative variance explained by the factors. The relevant analysis component included items with a loading value of 0.4 or higher (16).

Subsequently, confirmatory factor analysis (CFA) was applied to assess how many factors in the SIS 3.0 model provided the best explanation of stroke patients’ HRQoL. Using the Satorra-Bentler correction for non-normality, we estimated the fit of the observed data based on the respective cut-off values of various model fit indicators (17):

- Relative Chi-square (*χ*^2^/df ) : A value ≤ 3.0 indicates a good fit.
- Root Mean Square Error of Approximation (RMSEA): A value ≤ 0.08 indicates a good fit.
- Comparative Fit Index (CFI): A value ≥ 0.9 indicates an acceptable fit.
- Tucker-Lewis Index (TLI): A value ≥ 0.9 indicates an acceptable fit.
- Standardized Root Mean Square Residual (SRMR): A value ≤ 0.08 indicates a good fit.

#### 2.3.3 Convergent and divergent validity

The convergent and divergent validity of the V-SIS 3.0 scale was evaluated using Pearson’s correlation matrix. Convergent validity was considered insufficient if the diagonal values were below 0.4. Similarly, divergent validity was deemed insufficient if the off-diagonal values in each row exceeded the diagonal values.

#### 2.3.4 Item response theory (IRT)

IRT encompasses psychometric techniques for analyzing items, item responses, and overall scale characteristics. The core principle of IRT is that the likelihood of a given response is influenced by an underlying ability or trait, represented by Theta (*θ*), which lies on a continuous latent dimension. Theta represents an individual’s true underlying ability, standardized to a normal distribution ranging from −3.00 to 3.00 (18). IRT analyses allow for distinguishing item properties, such as discrimination and difficulty, across individuals over a broader range of the measured construct (19).

## 3 Results

A total of 256 participants were included in the final analysis, predominantly older adults with a mean age of 72.9 ± 10.2 years and a slight male predominance (52.7%). The sample was relatively homogeneous regarding age and stroke severity. Most individuals had ischemic strokes (55.1%), with the majority reporting a single stroke event (62.9%) and participating within 6 months post-stroke (30.9%). Overall, the average MRS score (2.5 ± 1.5) and SIS scores (48.0 ± 22.3 across domains) indicate moderate levels of disability and overall HRQoL. Further details on the socio-demographic and stroke-related characteristics, as well as SIS and MRS scores, are summarized in Table 1.

**Table 1:** Sociodemographic, stroke-related characteristics, and Vietnamese stroke impact scale 3.0 score (*n* = 256).

Characteristics	*n*	%
Sex		
Male	135	52.7
Female	121	47.3
Occupation		
Not working	151	59.0
Working	105	41.0
Education		
Secondary school or lower	124	48.4
High school or higher	132	51.6
Marital status		
Living with spouse	189	73.8
Single, widow, or widower	67	26.2
Stroke classification		
Ischemia	141	55.1
Hemorrhage	36	14.0
Unknown	79	30.9
Number of stroke onsets		
Single occurrence	161	62.9
Multiple occurrences	95	37.1
Time from stroke onset to study participation		
< 1 month	55	21.5
1 month to < 3 months	48	18.7
3 months to < 6 months	79	30.9
6 months to 1 year	74	28.9
Stroke side		
Right-sided	106	41.4
Left-sided	98	38.3
Unknown	52	20.3

Characteristics	Mean	SD
Age	72.9	10.2
Modified Rankin scale - MRS (0–5)	2.5	1.5
Stroke impact scale 3.0 - SIS (0–100)	48.0	22.3
Strength	52.0	26.8
Memory and thinking	44.7	29.0
Emotion	41.1	19.8
Communication	32.9	29.2
Activities of daily living	47.4	28.6
Mobility	55.8	32.8
Hand function	59.7	34.9
Participation	50.6	30.8

Scree parallel analysis and EFA both suggested that a four-factor model was the best fit for the V-SIS 3.0 (Figure 1). As shown in Table 2, most of the communality values ranged from moderate to high. The Kaiser-Meyer-Olkin (KMO) measure of sampling adequacy was 0.948, indicating that the sample was well-suited for EFA. Additionally, Bartlett’s Test of Sphericity showed a *p*-value < 0.001 (*χ*^2^ = 19900.640; degrees of freedom = 1711), confirming that the correlation matrix was appropriate for factor analysis. The EFA supported a four-factor solution, which comprised: factor 1, “Physical” (28 items); factor 2, “Cognitive” (12 items); factor 3, “Social Participation” (10 items); and factor 4, “Emotional” (8 items). Items with factor loadings below 0.4 were excluded from the final model, resulting in a more refined version compared to the original instrument.

**Table 2:** EFA of the stroke impact scale 3.0 using oblique rotation.

SIS 3.0 items	Factor 1: physical	Factor 2: cognitive	Factor 3: social participation	Factor 4: emotional
In the past week, how would you rate the strength of your…				
(1a) Arm that was most affected by your stroke?	0.7418			
(1b) Grip of your hand that was most affected by your stroke?	0.7341			
(1c) Leg that was most affected by your stroke?	0.7353			
(1d) Foot/ankle that was most affected by your stroke?	0.7342			
In the past week, how difficult was it for you to…				
(2a) Remember things that people just told you?		0.7986		
(2b) Remember things that happened yesterday?		0.8489		
(2c) Remember to do things (e.g., keep scheduled appointments or take medication)?		0.8202		
(2d) Remember the day of the week?		0.8266		
(2f) Concentrate?		0.8067		
(2g) Think quickly?		0.7609		
(2h) Solve problems?		0.7259		
In the past week, how often did you…				
(3a) Feel sad?				0.7337
(3b) Feel that there is nobody you are close to?				0.7562
(3c) Feel that you are a burden to others?				0.7492
(3d) Feel that you have nothing to look forward to?				0.7712
(3e) Blame yourself for mistakes?				0.7701
(3f) Enjoy things as much as you ever have?*				
(3g) Feel quite nervous?				0.5746
(3h) Feel that life is worth living?*				
(3i) Smile and laugh at least once a day?*				
In the past week, how difficult was it to…				
(4a) Say the name of someone whose face was in front of you?	0.7212			
(4b) Understand what was being said to you in a conversation?	0.7458			
(4c) Reply to questions?		0.7362		
(4d) Correctly name objects?		0.7248		
(4e) Participate in a conversation with a group of people?		0.7825		
(4f) Have a conversation on the telephone?		0.7339		
(4g) Call another person on the telephone (select the correct phone number and dial)?		0.7094		
In the past 2 weeks, how difficult was it to…				
(5a) Cut your food with a knife and fork?	0.6109			
(5b) Dress the top part of your body?	0.8008			
(5c) Bathe yourself?	0.8360			
(5d) Clip your toenails?	0.8079			
(5e) Get to the toilet on time?	0.7855			
(5f) Control your bladder (not have an accident)?	0.6111			
(5g) Control your bowels (not have an accident)?	0.6006			
(5h) Do light household tasks/chores (e.g., dust, make a bed, take out garbage, do the dishes)?	0.8148			
(5i) Go shopping?			0.4406	
(5j) Do heavy household chores (e.g., vacuum, laundry or yard work)?	0.7451			
In the past 2 weeks, how difficult was it to…				
(6a) Stay sitting without losing your balance?	0.6873			
(6b) Stay standing without losing your balance?	0.8638			
(6c) Walk without losing your balance?	0.8750			
(6d) Move from a bed to a chair?	0.8842			
(6e) Walk one block?	0.8731			
(6f) Walk fast?	0.8551			
(6g) Climb one flight of stairs?	0.8562			
(6h) Climb several flights of stairs?	0.8451			
(6i) Get in and out of a car?			0.4471	
In the past 2 weeks, how difficult was it to use your hand that was most affected by your stroke to…				
(7a) Carry heavy objects (e.g., bag of groceries)?	0.7904			
(7b) Turn a doorknob?	0.8087			
(7c) Open a can or jar?	0.7877			
(7d) Tie a shoelace?	0.8248			
(7e) Pick up a dime?	0.7823			
During the past 4 weeks, how much of the time have you been limited in…				
(8a) Your work, volunteer or other activities?			0.8435	
(8b) Your social activities?			0.8702	
(8c) Quiet recreation (crafts, reading)?			0.8035	
(8d) Active recreation (sports, outings, travel)?			0.8858	
(8e) Your role as a family member and/or friend?			0.6555	
(8f) Your participation in spiritual or religious activities?			0.7964	
(8h) Your ability to control your life as you wish?			0.7896	
(8i) Your ability to help others in need?			0.8686	

Table presents the factor loadings for each SIS 3.0 item across four domains—Physical, Cognitive, Social Participation, and Emotional—derived from an exploratory factor analysis using oblique rotation. Items are grouped according to their respective sections in the questionnaire, and only the highest loading per item is displayed. Items marked with an asterisk (*) indicate reverse-coded responses.

**Figure 2:**
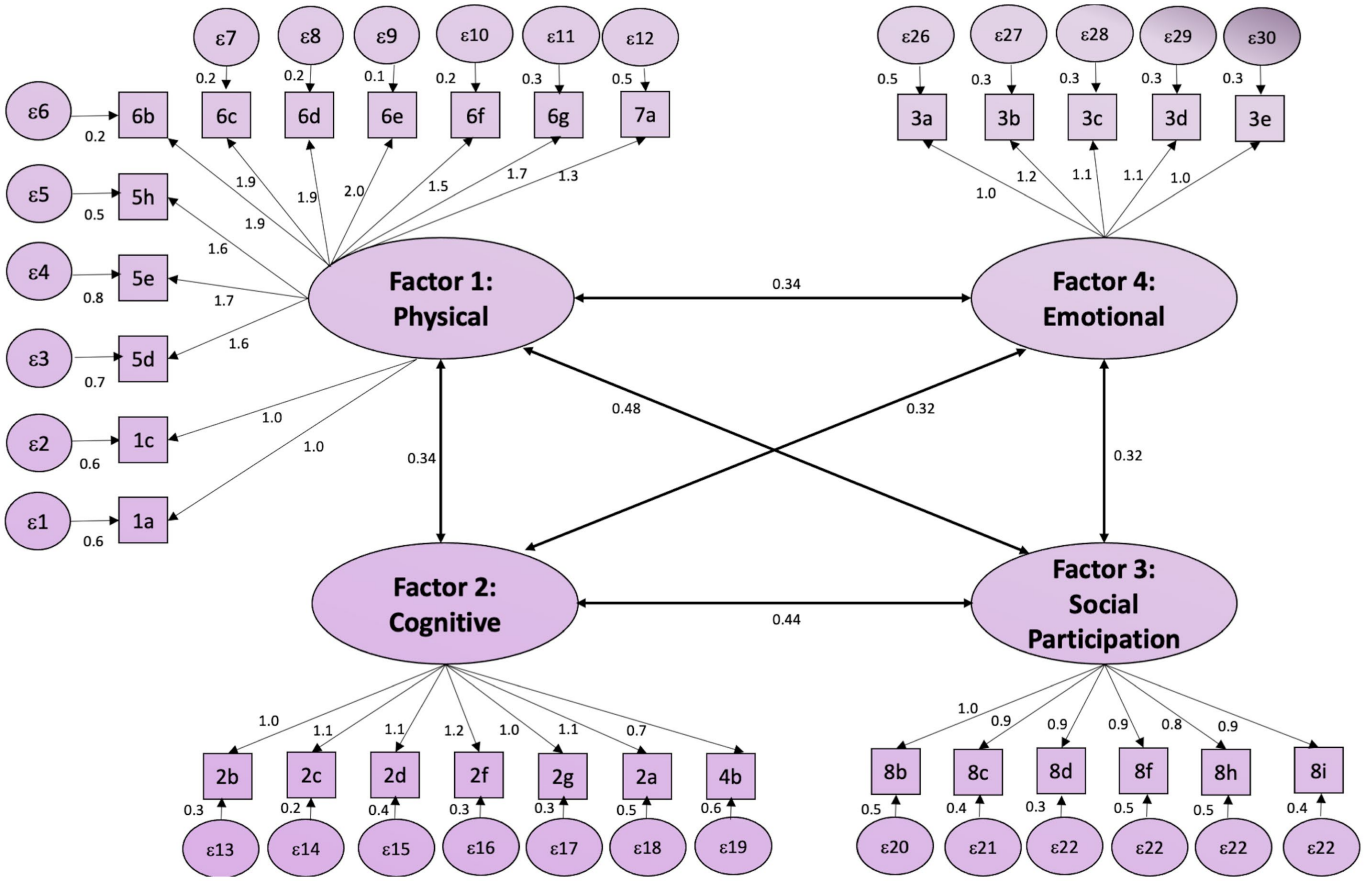
CFA model for the short-form stroke impact scale. The figure illustrates the CFA model for the Short-Form Stroke Impact Scale, comprising four latent factors: Physical, Cognitive, Social Participation, and Emotional. Each factor is represented as an oval, with observed variables (items) as rectangles. The arrows indicate the factor loadings and residual variances are shown as circles. Inter-factor correlations are displayed with bidirectional arrows and corresponding values, demonstrating the relationships among the latent factors.

CFA further supported this four-factor structure, with multiple fit indices meeting acceptable thresholds (Figure 2). To achieve a model with the goodness-of-fit indices (RMSEA [90% CI] = 0.080 [0.075, 0.088], CFI = 0.925, TLI = 0.918, and SRMR = 0.053 (*p* ≤ 0.001)), several items were removed. This process resulted in the final V-SIS 3.0 containing 29 items, each scored on a 1–5 scale (Table 3). Skewness values for these items ranged from −0.2 to 1.2, and Kurtosis values from 1.3 to 3.7, with 28 out of 29 items remaining below a Kurtosis of 3.0. Internal consistency was excellent, as indicated by Cronbach’s alpha values of 0.9713 (Physical), 0.9497 (Cognitive), 0.9494 (Social Participation), and 0.9195 (Emotional). Most items-to-factor correlations were also strong (r > 0.6).

**Table 3:** Basic descriptions and reliability of the short-form stroke impact scale.

Items	Responses (%)					Mean (SD)	Skewness	Kurtosis	Floor (%)	Ceiling (%)	Item total correlation	Cronbach’s alpha if item deleted
	1	2	3	4	5							
Factor 1: Physical												
(1a) Arm that was most affected by your stroke?	0.0	42.2	22.7	15.6	19.5	3.1 (1.2)	0.5	1.8	42.2	19.5	0.64	0.95
(1c) Leg that was most affected by your stroke?	0.0	44.5	23.4	14.8	17.2	3.0 (1.1)	0.6	1.9	44.5	17.2	0.67	0.95
(5d) Clip your toenails?	21.5	15.6	10.5	16.0	36.3	3.3 (1.6)	−0.2	1.5	21.5	36.3	0.79	0.95
(5e) Get to the toilet on time?	41.0	14.1	7.8	10.9	26.2	2.7 (1.7)	0.3	1.4	41.0	26.2	0.78	0.95
(5h) Do light household tasks/chores (e.g., dust, make a bed, take out garbage, do the dishes)?	16.8	12.9	11.7	14.8	43.8	3.6 (1.5)	−0.5	1.7	16.8	43.7	0.81	0.95
(6b) Stay standing without losing your balance?	23.0	15.6	13.3	11.7	36.3	3.2 (1.6)	−0.1	1.4	23.1	36.3	0.84	0.95
(6c) Walk without losing your balance?	21.1	16.0	10.9	13.3	38.7	3.3 (1.6)	−0.2	1.5	21.1	38.7	0.83	0.95
(6d) Move from a bed to a chair?	28.5	14.5	15.2	11.3	30.5	3.0 (1.6)	0.0	1.4	28.5	30.5	0.82	0.95
(6e) Walk one block?	27.3	14.5	11.3	10.5	36.3	3.1 (1.7)	−0.1	1.3	27.3	36.3	0.85	0.95
(6f) Walk fast?	4.7	21.9	12.1	15.2	46.1	3.8 (1.3)	−0.5	1.8	4.7	46.1	0.79	0.95
(7a) Carry heavy objects (e.g., bag of groceries)?	5.5	16.0	14.1	18.4	46.1	3.8 (1.3)	−0.7	2.1	5.5	46.1	0.76	0.95
Factor 2: Cognitive												
(2a) Remember things that people just told you?	28.9	23.8	27.3	14.8	5.1	2.4 (1.2)	0.4	2.1	28.9	5.1	0.61	0.95
(2b) Remember things that happened yesterday?	15.6	31.6	21.9	20.3	10.5	2.8 (1.2)	0.2	2.0	15.6	10.6	0.57	0.95
(2c) Remember to do things (e.g., keep scheduled appointments or take medication)?	16.0	24.2	21.5	22.3	16.0	3.0 (1.3)	0.0	1.8	16.0	16.0	0.58	0.95
(2d) Remember the day of the week?	22.3	23.4	17.2	23.0	14.1	2.8 (1.4)	0.1	1.7	22.3	14.1	0.63	0.95
(2f) Concentrate?	19.5	26.6	26.6	18.0	9.4	2.7 (1.2)	0.2	2.1	19.5	9.4	0.62	0.95
(2g) Think quickly?	24.6	20.3	20.3	19.9	14.8	2.8 (1.4)	0.1	1.7	24.6	14.8	0.59	0.95
(4b) Understand what was being said to you in a conversation?	47.3	28.9	13.3	5.9	4.7	1.9 (1.1)	1.2	3.7	47.3	4.7	0.62	0.95
Factor 3: social participation												
(8b) Your social activities?	23.0	16.0	17.2	19.1	24.6	3.0 (1.5)	−0.1	1.6	23.0	24.6	0.62	0.95
(8c) Quiet recreation (crafts, reading)?	19.5	26.2	18.8	20.3	15.2	2.9 (1.4)	0.1	1.8	19.5	15.2	0.62	0.95
(8d) Active recreation (sports, outings, travel)?	14.1	22.3	19.5	21.5	22.7	3.2 (1.4)	−0.1	1.7	14.1	22.7	0.55	0.95
(8f) Your participation in spiritual or religious activities?	28.9	19.5	19.1	14.8	17.6	2.7 (1.5)	0.3	1.7	28.9	17.6	0.66	0.95
(8h) Your ability to control your life as you wish?	14.1	16.0	25.4	26.2	18.4	3.2 (1.3)	−0.2	2.0	14.1	18.4	0.56	0.95
(8i) Your ability to help others in need?	15.2	17.2	19.1	22.7	25.8	3.3 (1.4)	−0.2	1.8	15.2	25.8	0.57	0.95
Factor 4: Emotional												
(3a) Feel sad?	13.3	36.3	26.2	20.7	3.5	2.6 (1.1)	0.2	2.2	13.3	3.5	0.54	0.95
(3b) Feel that there is nobody you are close to?	32.0	27.7	22.3	15.2	2.7	2.3 (1.1)	0.5	2.1	32.0	2.7	0.58	0.95
(3c) Feel that you are a burden to others?	30.9	28.5	21.5	16.8	2.3	2.3 (1.1)	0.4	2.1	30.9	2.3	0.58	0.95
(3d) Feel that you have nothing to look forward to?	39.8	27.3	18.0	12.9	2.0	2.1 (1.1)	0.7	2.4	39.8	1.9	0.55	0.95
(3e) Blame yourself for mistakes?	48.4	22.7	18.4	9.0	1.6	1.9 (1.1)	0.9	2.7	48.4	1.6	0.48	0.95

Table displays response distributions (percentages across a five-point scale), mean scores with standard deviations, skewness, kurtosis, floor and ceiling effects, item-total correlations, and Cronbach’s alpha values if the item is deleted. Items are organized by domain (Physical, Cognitive, Social Participation, and Emotional) to illustrate their individual performance and contribution to the overall scale reliability.

**Figure 3:**
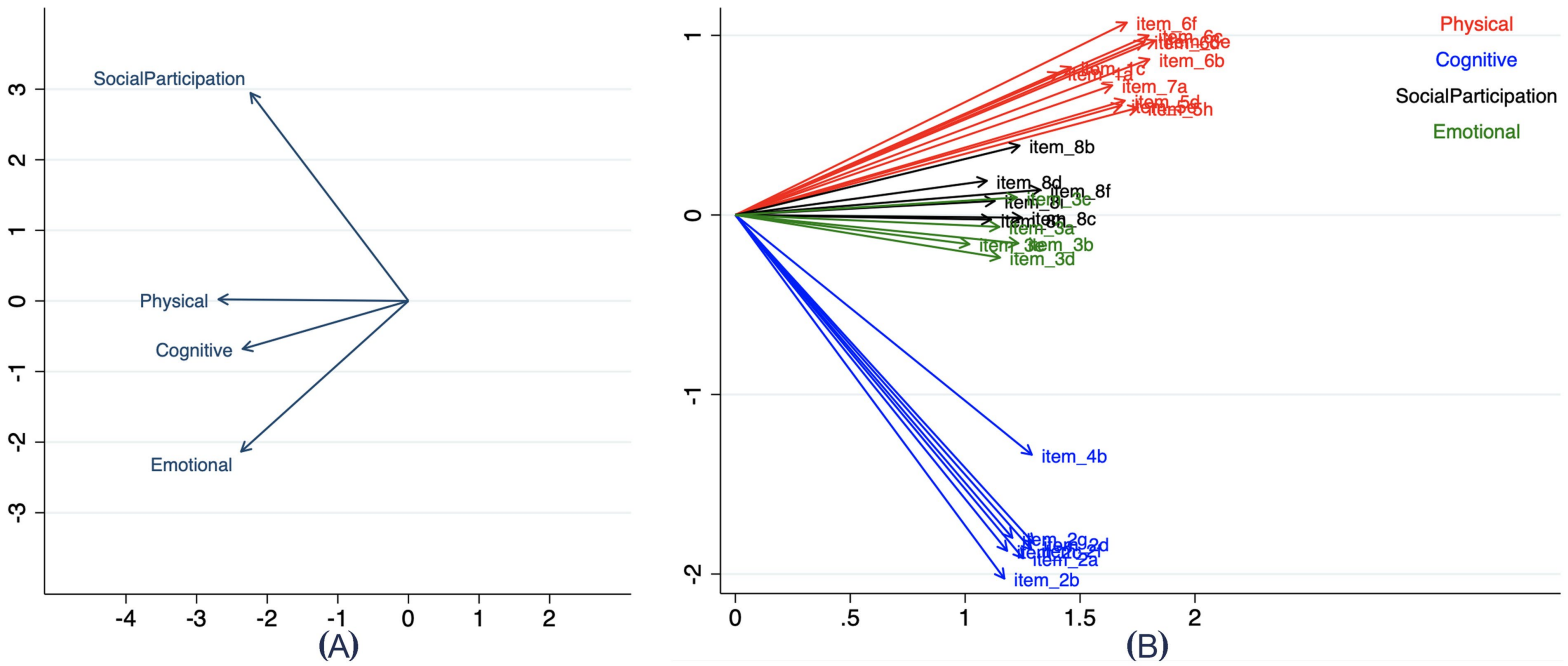
Correlation between domains **(A)** and items **(B)**. **(A)** illustrates the correlations among the four domains: Physical, Cognitive, Social Participation, and Emotional. **(B)** displays the item-level correlations, with each arrow representing the relationship between individual items and their corresponding domains. Items are color-coded according to their domain for clarity: red for Physical, blue for Cognitive, green for Emotional, and black for Social Participation. These visualizations highlight the relationships within and between domains and their respective items.

**Figure 4:**
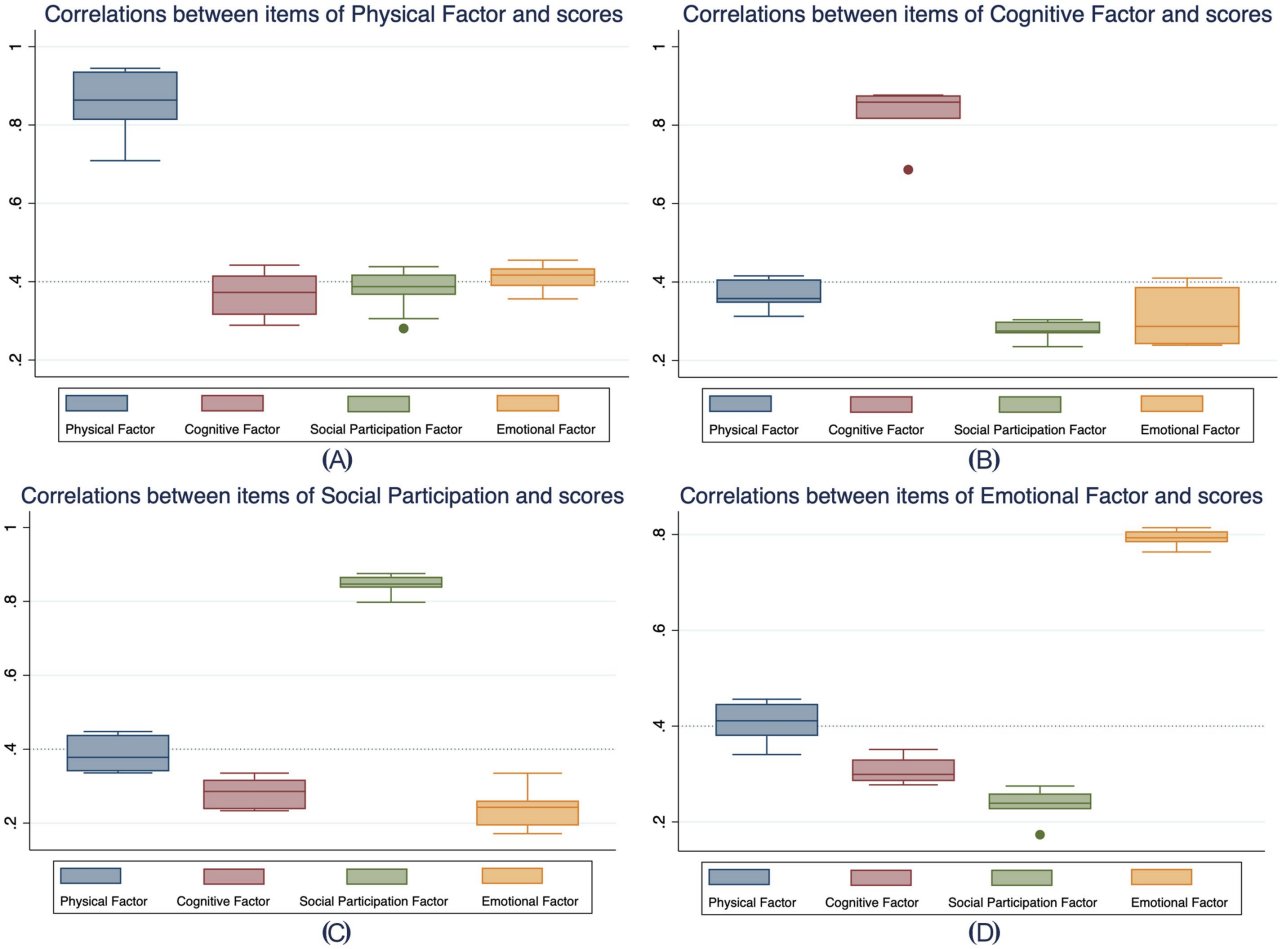
Correlations between items within each domain and scores of other domains. **(A–D)** illustrate the correlations between items within the Physical, Cognitive, Social Participation, and Emotional domains, respectively, and the scores of all four domains. Each box plot represents the range, median, and variability of item correlations within each domain (color-coded) in relation to the target domain and the other three domains. This visualization highlights the strength of associations within domains and cross-domain correlations, aiding in the evaluation of domain-specific and cross-domain relationships.

The correlation patterns among subscales and items of the final V-SIS 3.0 are depicted in Figure 3. Figure 3A demonstrates negative correlations between the Physical and Cognitive subscales and the Emotional and Social Participation subscales, while Figure 3B highlights stronger intercorrelations within each subscales. Boxplots in Figure 4 offer additional evidence of convergent and divergent validity by illustrating that items within the same subscale correlate more highly with each other than with items in other subscales.

As detailed in Table 4, convergent validity was satisfactory, with all items correlating above 0.4 with their respective factor scores. Divergent validity was similarly supported by each item showing a higher correlation with its own factor score than with the other factor scores. Finally, Supplementary Table 2 reports high discrimination values across all items (ranging from 2.03 to 9.77 for the Physical factor, and 0.93 to 1.35, 1.03 to 1.27, and 0.95 to 1.15 for the Cognitive, Social Participation, and Emotional factors, respectively). Most items fell within typical difficulty parameters (−2 to +2), although seven items (2a, 2b, 3a, 3b, 3c, 3d, and 3e) exceeded a difficulty parameters of 3 for the last response exceeded option, indicating that only participants with very high latent trait levels would endorse them.

**Table 4:** Evaluating convergent and divergent validity using the correlation matrix of items and domain scores.

Items	Factor 1: physical	Factor 2: cognitive	Factor 3: social participation	Factor 4: emotional
Factor 1: Physical				
(1a) Arm that was most affected by your stroke?	**0.7609**	0.3569	0.3186	0.3602
(1c) Leg that was most affected by your stroke?	**0.7643**	0.3624	0.3387	0.3872
(5d) Clip your toenails?	**0.8726**	0.5130	0.4243	0.3859
(5e) Get to the toilet on time?	**0.8712**	0.4952	0.4013	0.4188
(5h) Do light household tasks/chores (e.g., dust, make a bed, take out garbage, do the dishes)?	**0.8812**	0.5227	0.4250	0.4190
(6b) Stay standing without losing your balance?	**0.9191**	0.4760	0.4491	0.4209
(6c) Walk without losing your balance?	**0.9205**	0.4495	0.4473	0.4444
(6d) Move from a bed to a chair?	**0.9265**	0.4631	0.4115	0.4225
(6e) Walk one block?	**0.9274**	0.4620	0.4621	0.4452
(6f) Walk fast?	**0.8798**	0.4080	0.4587	0.3695
(7a) Carry heavy objects (e.g., bag of groceries)?	**0.8409**	0.4495	0.4287	0.3959
Factor 2: Cognitive				
(2a) Remember things that people just told you?	0.4027	**0.8359**	0.2547	0.3673
(2b) Remember things that happened yesterday?	0.3492	**0.8455**	0.3212	0.2376
(2c) Remember to do things (e.g., keep scheduled appointments or take medication)?	0.3742	**0.8267**	0.3094	0.2462
(2d) Remember the day of the week?	0.4368	**0.8500**	0.3229	0.2678
(2f) Concentrate?	0.4359	**0.8513**	0.2491	0.3840
(2g) Think quickly?	0.3879	**0.7977**	0.2610	0.2822
(4b) Understand what was being said to you in a conversation?	0.4674	**0.8277**	0.3227	0.4183
Factor 3: Social participation				
(8b) Your social activities?	0.4192	0.2644	**0.8894**	0.2403
(8c) Quiet recreation (crafts, reading)?	0.4092	0.3568	**0.8490**	0.2649
(8d) Active recreation (sports, outings, travel)?	0.3267	0.2519	**0.8952**	0.1986
(8f) Your participation in spiritual or religious activities?	0.4351	0.3396	**0.8483**	0.3363
(8h) Your ability to control your life as you wish?	0.3353	0.2809	**0.8095**	0.2408
(8i) Your ability to help others in need?	0.3352	0.2811	**0.8719**	0.1779
Factor 4: Emotional				
(3a) Feel sad?	0.4400	0.3539	0.2390	**0.8494**
(3b) Feel that there is nobody you are close to?	0.4790	0.3944	0.1969	**0.8599**
(3c) Feel that you are a burden to others?	0.4761	0.3544	0.2632	**0.8698**
(3d) Feel that you have nothing to look forward to?	0.4056	0.3743	0.2267	**0.8646**
(3e) Blame yourself for mistakes?	0.3714	0.3270	0.1541	**0.8555**

Table presents the correlation coefficients between individual items and the four domain scores—Physical, Cognitive, Social Participation, and Emotional. Higher correlations between an item and its corresponding domain (convergent validity) compared to correlations with other domains (divergent validity) support the distinctiveness of the scale’s constructs. Note: Bold values indicate the highest correlation of each item with its corresponding domain (i.e., convergent validity), demonstrating that each item is most strongly associated with the factor it is intended to measure.

## 4 Discussion

The present study aimed to adapt and evaluate the psychometric properties of the V-SIS 3.0, providing valuable insights for integrated strategies that promote lifelong health among stroke survivors. By refining the original eight-domain structure into a more parsimonious four-factor model—comprising Physical, Cognitive, Social Participation, and Emotional domains—we achieved excellent internal consistency (Cronbach’s alpha > 0.91) while enhancing the scale’s efficiency and clinical utility. These results highlight the potential of culturally tailored measurement tools to support multidimensional approaches to older adult stroke survivors in Vietnam.

The transition from an eight-domain to a four-domain structure highlights a critical consideration in health assessment: the balance between comprehensive evaluation and practical applicability. The robust psychometric properties of the refined V-SIS 3.0 suggest that essential aspects of HRQoL in stroke survivors can be captured with fewer items, facilitating quicker assessments without compromising reliability or validity. This efficiency is particularly relevant for integrated lifelong health strategies, where routine monitoring and rapid feedback can empower clinicians and patients alike to adjust interventions in real-time.

The strong convergent and divergent validity observed in our study not only reinforces the scale’s measurement integrity but also aligns with the broader paradigm of multidimensional health assessment. In the context of aging and lifestyle interventions, precise and culturally relevant measurement tools are indispensable. They enable healthcare providers to identify specific areas—be it physical, cognitive, social, or emotional—that require targeted intervention, thereby fostering a holistic approach to rehabilitation and health promotion.

Convergent validity is a critical consideration in psychometric evaluations. Ideally, moderate correlations between instruments assessing similar constructs support validation (20). However, high correlations may still be acceptable if the validated instrument offers advantages such as improved brevity or ease of administration (20). Our study found that the four factors exhibited relatively high correlations, with the “Physical” and “Emotional” domains contributing slightly more to HRQoL than the “Cognitive” and “Social Participation” domains. Moreover, each item’s correlation coefficient was stronger with its assigned factor than with other factors, reinforcing the scale’s divergent validity (20). Additionally, individuals reporting significant functional impairments had substantially lower scores across all factors, supporting the scale’s discriminant validity.

The psychometric properties of SIS 3.0 have been extensively evaluated across various cultural and linguistic contexts, with findings comparable to those of our study. Previous research in the United States and Europe has consistently demonstrated high internal consistency across the original eight-factor structure, with Cronbach’s alpha values exceeding 0.80 (13, 21). Similar to our findings, these studies have reported strong convergent and divergent validity, affirming the SIS 3.0 as a reliable tool for assessing post-stroke HRQoL. However, adaptations of the SIS 3.0 in different populations have sometimes required modifications to improve model fit, as seen in studies from Brazil, where factor structures were adjusted to enhance validity and cultural relevance (12). The study by Vellone et al. (21) in Italy showed a similar finding to our research, which identified a refined four-factor structure that demonstrated superior psychometric properties compared to the original eight-factor model. In our study, the transition to a four-factor structure in the V-SIS 3.0 aligns with these findings, highlighting the importance of cultural and contextual adaptations in psychometric evaluations. However, a study in Taiwan concluded that the eight- and four-domain scores of the SIS 3.0 may not be valid (22). Therefore, until further supporting evidence is available, these scores should be interpreted cautiously when assessing patients’ HRQOL, and alternative measures may be considered. Despite variations in factor structures, the consistently high internal consistency and validity reported across studies reinforce the robustness of SIS 3.0 as an assessment tool. Future research should continue to explore cross-cultural differences to enhance the instrument’s applicability in diverse populations.

To our knowledge, this study represents the first effort in Vietnam to translate and evaluate the psychometric properties of SIS 3.0. Sample size considerations are important in scale validation research, with recommendations suggesting that instruments with fewer than 20 items require 100–200 participants for reliable psychometric assessment (23). Our study utilized a sample size of 256, which is adequate for evaluating the psychometric properties of the 29-item V-SIS 3.0. Importantly, this validation was conducted in a hospital setting, which may influence its generalizability. However, our sample included stroke survivors with varying levels of severity, enhancing the applicability of our findings to a broader stroke population. Additionally, while the psychometric analysis was performed during the chronic phase of stroke, the interval between stroke onset and study participation did not appear to influence item performance significantly. These findings suggest that the V-SIS 3.0 is a stable and reliable tool for assessing HRQoL in Vietnamese stroke survivors.

Incorporating the V-SIS 3.0 into clinical practice has several implications for lifelong health strategies. First, the ability to rapidly and accurately assess HRQoL can inform personalized rehabilitation programs, ensuring that interventions address the most impactful domains for individual stroke survivors. For instance, our findings indicate that the Physical and Emotional domains are particularly influential in overall HRQoL. Tailoring interventions to bolster physical recovery and emotional wellbeing could, therefore, yield substantial benefits in post-stroke care. Moreover, the streamlined nature of the V-SIS 3.0 makes it a suitable candidate for integration into larger multidimensional frameworks that include lifestyle modifications, preventive health measures, and chronic disease management. In a broader aging population, similar strategies that emphasize early detection of declines in HRQoL, followed by tailored lifestyle interventions, could promote resilience, prolong independence, and ultimately enhance quality of life.

Despite the strengths of this study, several limitations should be acknowledged. First, while EFA and CFA were instrumental in refining the structure of the V-SIS 3.0, EFA can be influenced by subjective item selection and factor retention criteria. The potential for bias in factor determination underscores the need for future studies to explore alternative retention methods, use larger and more diverse samples, and perform cross-validation with independent datasets to enhance generalizability. Second, we did not compare the V-SIS 3.0 against other widely used HRQoL scales, such as those measuring disability or activities of daily living. Incorporating such comparisons in future studies would provide a more comprehensive contextualization of our findings and further validate the scale’s utility in assessing HRQoL in stroke survivors. Third, while our study included stroke survivors with varying severity levels, we did not formally evaluate the scale’s metric properties across different severity subgroups. Future research should investigate whether the V-SIS 3.0 maintains its reliability and validity across mild, moderate, and severe stroke populations to ensure its broad applicability. Lastly, the cross-sectional nature of our study limits our ability to infer causality or track changes in HRQoL over time. Longitudinal studies should be conducted to explore how V-SIS 3.0 scores fluctuate during the stroke recovery process and how they relate to long-term rehabilitation outcomes.

## 5 Conclusion

This study provides compelling evidence that the V-SIS 3.0 is a reliable and valid tool for assessing HRQoL in Vietnamese stroke survivors. The refined four-factor model—encompassing physical, cognitive, emotional, and social wellbeing—demonstrates robust internal consistency and validity, offering an efficient structure for clinical evaluations and research applications. Importantly, by capturing multiple dimensions of health, the V-SIS 3.0 supports integrated strategies for lifelong health, aging, and lifestyle interventions of stroke survivors. Its multidimensional framework not only enhances the precision of stroke rehabilitation assessments but also informs the design of targeted interventions that can adapt to the evolving needs of stroke survivors over time. Despite certain limitations, the scale’s ability to provide a comprehensive view of HRQoL is a valuable asset in promoting practical, personalized, and sustainable health strategies for older adult stroke survivors in Vietnam.

## Data Availability

The raw data supporting the conclusions of this article will be made available by the authors, without undue reservation.
