# Supplementary material for: Optimizing health-related quality of life assessments for stroke survivors: a validation study of psychometric properties for the Vietnamese version of stroke impact scale 3.0
Source: Front Public Health. 2025 May 20;13:1570980. doi: 10.3389/fpubh.2025.1570980 (PMC12129923; doi:10.3389/fpubh.2025.1570980)
Supplement: Supplementary file 1 [file Table_1.docx]

**Supplementary 1. Key Differences and Cultural Adaptations between the Original SIS 3.0 (English) and the Adapted SIS 3.0 (Vietnamese)**

| **Item / Domain** | **Original SIS 3.0 (English)** | **Adapted SIS 3.0 (Vietnamese)** | **Rationale for Cultural Adaptation** |
| --- | --- | --- | --- |
| **1. Strength Domain (Items 1.1–1.4)** | | | |
| **Instruction** | “In the past week, how would you rate the strength of your…” | “Trong tuần qua, ông/bà đánh giá thế nào về sức lực/sức mạnh của…” | Added both “sức lực” (strength) and “sức mạnh” (power) for clearer meaning in Vietnamese. |
| **1.1** | “Arm that was most affected by your stroke?” | “Bên cánh tay bị ảnh hưởng nhiều nhất sau đột quỵ của ông/bà?” | Replaced “arm … most affected by your stroke” with a more explicit phrase indicating the side of the arm. |
| **1.2** | “Grip of your hand that was most affected by your stroke?” | “Việc nắm bàn tay của ông/bà bị ảnh hưởng nhiều nhất sau đột quỵ?” | Clarifies “grip” to “việc nắm bàn tay” (“the act of gripping with the hand”). |
| **1.3** | “Leg that was most affected by your stroke?” | “Bên chân bị ảnh hưởng nhiều nhất sau đột quỵ của ông/bà?” | Straightforward translation; “bên chân” clarifies which side. |
| **1.4** | “Foot/ankle that was most affected by your stroke?” | “Bàn chân/mắt cá chân bị ảnh hưởng nhiều nhất sau đột quỵ của ông/bà?” | Added both “bàn chân” (foot) and “mắt cá chân” (ankle) to match original item. |
| **Rating Scale** | Typically: 5 = “A lot of strength,” 4 = “Quite a bit of strength,” … 1 = “No strength at all.” | 5 = “Rất nhiều sức lực,” 4 = “Khá nhiều sức lực,” … 1 = “Hoàn toàn không có chút sức lực.” | Minor wording changes. Ensures each level of strength is culturally and linguistically clear (e.g., “rất nhiều sức lực” for “a lot of strength”). |
| **2. Memory/Thinking Domain (Items 2.1–2.7)** | | | |
| **Instruction** | “In the past week, how much of a problem did you have… (e.g., remembering things that people just told you?)” | “Trong tuần vừa qua, ông/bà gặp khó khăn như thế nào khi… (ví dụ: ghi nhớ lại những điều mà mọi người vừa nói với ông/bà?)” | Direct translation of instructions, but rephrased to “gặp khó khăn như thế nào” (“how much difficulty”) for clarity. |
| **2.1** | “Remembering things that people just told you?” | “Ghi nhớ lại những điều mà mọi người vừa nói với ông/bà?” | Smooth adaptation. “Just told you” → “vừa nói với ông/bà.” |
| **2.2** | “Remembering things that happened the day before?” | “Ghi nhớ lại những điều đã xảy ra vào 1 ngày trước đó?” | Faithful translation. |
| **2.3** | “Remembering to do things (e.g., keep scheduled appointments, take medication)?” | “Ghi nhớ lại những việc (ví dụ: giữ các cuộc hẹn đã lên lịch hoặc uống thuốc)?” | Slight expansion: “giữ các cuộc hẹn đã lên lịch” (keep scheduled appointments) clarifies the concept. |
| **2.4** | “Remembering the day of the week?” | “Ghi nhớ được ngày trong tuần?” | Straightforward. |
| **2.5** | “Concentrating?” | “Tập trung?” | Direct translation, minor adjustment to verb form. |
| **2.6** | “Thinking quickly?” | “Ghi nhớ nhanh?” | Adapted to “ghi nhớ nhanh” (quick memory/recall). Some versions might use “suy nghĩ nhanh” for “thinking quickly.” |
| **2.7** | “Solving everyday problems?” | “Giải quyết các vấn đề hàng ngày?” | Direct equivalent. |
| **Rating Scale** | Typically: 5 = “No difficulty at all,” 4 = “A little difficulty,” … 1 = “Extremely difficult.” | 5 = “Hoàn toàn không khó khăn,” 4 = “Có một ít khó khăn,” … 1 = “Cực kỳ khó khăn.” | Terms adapted to reflect natural Vietnamese usage (e.g., “hoàn toàn không khó khăn” for “no difficulty at all”). |
| **3. Mood/Emotions Domain (Items 3.1–3.9)** | | | |
| **Instruction** | “In the past week, how often did you feel…” | “Trong tuần qua, tần suất ông/bà…” | Direct translation, uses “tần suất” (“frequency”). |
| **3.1** | “Sad?” | “Cảm thấy buồn” | Straightforward. |
| **3.2** | “That there was nobody you could turn to?” | “Cảm thấy không có ai ở bên cạnh?” | Reworded to a common Vietnamese expression for “no one around you / no support.” |
| **3.3** | “That you are a burden to others?” | “Cảm thấy bản thân là gánh nặng cho người khác?” | Faithful translation. |
| **3.4** | “That you had nothing to look forward to?” | “Cảm thấy bản thân không có điều gì để tiếp tục hướng về phía trước?” | Slightly more explicit, ensuring cultural clarity (feeling hopeless about the future). |
| **3.5** | “Blame yourself for mistakes that you made?” | “Tự trách mình vì những sai lầm mà bản thân đã gây ra?” | Straight translation. |
| **3.6** | “Enjoyed things as much as ever?” | “Tận hưởng mọi thứ nhiều hơn bao giờ hết?” | Subtle shift from “as much as ever” → “nhiều hơn bao giờ hết” (“more than ever”), which is a common Vietnamese phrasing to capture that sense of enjoyment. |
| **3.7** | “Felt quite nervous?” | “Cảm thấy khá lo lắng?” | Direct phrasing; “khá lo lắng” captures “quite nervous.” |
| **3.8** | “Felt that life was worth living?” | “Cảm thấy rằng cuộc sống là đáng sống?” | Straightforward. |
| **3.9** | “Smiled and laughed at least once a day?” | “Mỉm cười và cười lớn ít nhất một lần một ngày?” | Specifies both “mỉm cười” (smile) and “cười lớn” (laugh). |
| **Rating Scale** | Typically: 5 = “Never,” 4 = “Sometimes,” … 1 = “Always.” | 5 = “Không bao giờ,” 4 = “Thỉnh thoảng,” … 1 = “Mọi lúc.” | Aligned to Vietnamese usage: “Không bao giờ,” “Thỉnh thoảng,” “Mọi lúc,” etc. |
| **4. Communication Domain (Items 4.1–4.7)** | | | |
| **Instruction** | “In the past week, how difficult was it to…” | “Trong tuần qua, mức độ khó khăn như thế nào để…” | Direct translation. |
| **4.1** | “Say the name of someone who was in front of you?” | “Nói tên một ai đó đang đứng trước mặt bạn?” | Faithful translation. |
| **4.2** | “Understand what was being said to you in a conversation?” | “Hiểu những điều đang được nói với bạn trong một cuộc trò chuyện?” | Direct. |
| **4.3** | “Answer questions?” | “Trả lời câu hỏi?” | Straightforward. |
| **4.4** | “Name objects correctly?” | “Đặt tên chính xác cho các đồ vật?” | Faithful translation. |
| **4.5** | “Participate in a conversation with a group of people?” | “Tham gia vào cuộc trò chuyện với một nhóm người?” | Direct. |
| **4.6** | “Have a conversation on the telephone?” | “Nói chuyện qua điện thoại?” | Simplified phrasing. |
| **4.7** | “Call another person on the telephone, including selecting the correct number and dialing?” | “Gọi cho người khác qua điện thoại, bao gồm chọn số điện thoại chính xác và bấm số?” | Expanded to clarify the actions of choosing the correct number and dialing. |
| **Rating Scale** | Typically: 5 = “Not difficult at all,” 4 = “A little difficult,” … 1 = “Extremely difficult.” | 5 = “Hoàn toàn không khó khăn,” 4 = “Có một ít khó khăn,” … 1 = “Cực kỳ khó khăn.” | Same adaptation as Memory domain, using “hoàn toàn không khó khăn,” “cực kỳ khó khăn,” etc. |
| **5. ADL/IADL Domain (Items 5.1–5.10)** | | | |
| **Instruction** | “In the past 2 weeks, how difficult was it to…” | “Trong 2 tuần qua, mức độ khó khăn như thế nào để…” | Direct translation. |
| **5.1** | “Cut your food with a knife and fork?” | “Gắp/múc thức ăn bằng đũa/thìa?” | Adapted utensils to culturally relevant “đũa/thìa” (chopsticks/spoon) instead of “knife and fork.” |
| **5.2** | “Dress the top part of your body?” | “Tự mặc quần áo cho phần trên của cơ thể?” | Added “tự” (“by oneself”) for clarity. |
| **5.3** | “Bathe yourself?” | “Tự tắm rửa?” | Minor addition of “tự” for self-care emphasis. |
| **5.4** | “Clip your toenails?” | “Tự cắt móng chân?” | Direct translation plus “tự.” |
| **5.5** | “Control your bladder (not have an accident)?” | “Tự đi vệ sinh đúng giờ?” | Slight adaptation: “đi vệ sinh đúng giờ” is a softer expression for maintaining continence. |
| **5.6** | “Control your bladder?” (alternative phrasing in some SIS versions) | “Kiểm soát được việc đi tiểu (không bị són tiểu hoặc tiểu không tự chủ)?” | More explicit about potential incontinence. |
| **5.7** | “Control your bowels?” | “Kiểm soát được việc đại tiện (không bị đại tiện không tự chủ)?” | Same approach: clarifies incontinence. |
| **5.8** | “Do light household tasks (e.g., dusting)?” | “Làm các công việc/việc nhà nhẹ nhàng (ví dụ: quét nhà, dọn giường, đổ rác, rửa bát)?” | Expanded examples for culturally typical light household tasks (sweeping, washing dishes). |
| **5.9** | “Go shopping?” | “Đi chợ/mua sắm?” | “Đi chợ” (go to the market) is a culturally specific phrase. |
| **5.10** | “Do heavy household chores (e.g., vacuuming, yard work)?” | “Làm các công việc nhà nặng nhọc (ví dụ: hút bụi, giặt ủi, hay công việc ngoài sân)?” | Included tasks that are more common in Vietnamese contexts (yard work, laundry) alongside vacuuming. |
| **Rating Scale** | Typically: 5 = “Not difficult at all,” 4 = “A little difficult,” … 1 = “Could not do at all.” | 5 = “Hoàn toàn không khó khăn,” 4 = “Có một ít khó khăn,” … 1 = “Không thể thực hiện được.” | Final rating choice “Không thể thực hiện được” = “Could not do at all.” |
| **6. Mobility Domain (Items 6.1–6.9)** | | | |
| **Instruction** | “In the past 2 weeks, how difficult was it to…” | “Trong 2 tuần qua, mức độ khó khăn như thế nào để…” | Direct translation. |
| **6.1** | “Sit without losing your balance?” | “Ngồi mà không bị mất thăng bằng?” | Direct translation; no cultural change needed. |
| **6.2** | “Stand without losing your balance?” | “Đứng yên mà không bị mất thăng bằng?” | Adds “đứng yên” (“stand still”). |
| **6.3** | “Walk without losing your balance?” | “Đi bộ mà không bị mất thăng bằng?” | Straight translation. |
| **6.4** | “Transfer from a bed to a chair?” | “Di chuyển từ giường sang ghế?” | Direct translation. |
| **6.5** | “Walk one block?” (distance references can vary in some SIS versions) | “Bước đi một đoạn” | Adapted to “một đoạn” (a short distance) instead of specifically “one block,” which may not be universally understood in Vietnam. |
| **6.6** | “Walk fast?” | “Bước đi nhanh” | Straight translation. |
| **6.7** | “Climb one flight of stairs?” | “Leo lên một bậc thang?” | Adapted from “one flight of stairs” to “một bậc thang” or “một đoạn cầu thang.” The sample text uses “một bậc thang,” typically a single step. |
| **6.8** | “Climb several flights of stairs?” | “Leo lên vài bậc thang?” | Similarly adapted, acknowledging multi-step scenario. |
| **6.9** | “Get in and out of a car?” | “Tự bước lên hoặc xuống ô tô?” | Added “tự” and used “ô tô” (common Vietnamese term for car). |
| **Rating Scale** | Same as ADL domain (5 = “Not difficult at all,” … 1 = “Could not do at all”). | 5 = “Hoàn toàn không khó khăn,” … 1 = “Không thể thực hiện được.” | Consistent adaptation across all functional domains. |
| **7. Hand Function Domain (Items 7.1–7.5)** | | | |
| **Instruction** | “In the past 2 weeks, how difficult was it to use your hand that was most affected by the stroke to…” | “Trong 2 tuần qua, ông/bà gặp khó khăn như thế nào khi sử dụng bàn tay mà bị ảnh hưởng nhiều nhất sau đột quỵ để…” | Clarifies “sử dụng bàn tay… bị ảnh hưởng nhiều nhất” (“use the hand most affected by the stroke”). |
| **7.1** | “Carry heavy objects (e.g., bag of groceries)?” | “Mang các vật nặng (ví dụ: túi đựng đồ tạp hóa)?” | Faithful translation. |
| **7.2** | “Turn a doorknob?” | “Xoay nắm cửa?” | Direct. |
| **7.3** | “Open a can or jar?” | “Mở nắp chai hoặc lon?” | Adapted to reflect typical Vietnamese usage—“nắp chai hoặc lon” often more common than “jar.” |
| **7.4** | “Tie a shoe lace?” | “Buộc dây giày?” | Direct translation. |
| **7.5** | “Pick up a coin?” | “Nhặt một đồng xu?” | Straight translation; “đồng xu” clarifies it’s a coin. |
| **Rating Scale** | Same as ADL domain (5 = “Not difficult at all,” 4 = “A little difficult,” … 1 = “Could not do at all”). | 5 = “Hoàn toàn không khó khăn,” 4 = “Có một ít khó khăn,” … 1 = “Không thể thực hiện được.” | Consistent. |
| **8. Participation Domain (Items 8.1–8.8)** | | | |
| **Instruction** | “In the past 4 weeks, how much of the time have you been limited in…” | “Trong 4 tuần qua, ông/bà đã bị giới hạn bao nhiêu thời gian trong…” | Direct instruction, ensuring “bị giới hạn bao nhiêu thời gian” conveys “how much of the time.” |
| **8.1** | “Your work (paid, voluntary, or other)?” | “Công việc của ông/bà (được trả tiền, tình nguyện hoặc khác)?” | Straight translation. |
| **8.2** | “Your social activities?” | “Các hoạt động xã hội của bạn?” | Direct. |
| **8.3** | “Quiet recreational activities (crafts, reading)?” | “Hoạt động thư giãn yên tĩnh (làm đồ thủ công, đọc sách)?” | Added “làm đồ thủ công” to parallel “crafts.” |
| **8.4** | “Active recreational activities (sports, outings, travel)?” | “Hoạt động giải trí vận động (thể thao, đi chơi, du lịch)?” | “Hoạt động giải trí vận động” used to convey “active recreation.” |
| **8.5** | “Your role as a family member and/or friend?” | “Vai trò của bạn như một thành viên trong gia đình và/hoặc bạn bè?” | Slight rephrasing. |
| **8.6** | “Your spiritual or religious activities?” | “Sự tham gia của ông/bà vào các hoạt động tâm linh hay tôn giáo?” | “Các hoạt động tâm linh hay tôn giáo” is a common phrase in Vietnamese. |
| **8.7** | “Your ability to control your life as you wish?” | “Khả năng để kiểm soát cuộc sống của ông/bà như bản thân mong muốn?” | More explicit reference to personal autonomy and desire. |
| **8.8** | “Your ability to help others?” | “Khả năng giúp đỡ người khác của bản thân?” | Straightforward. |
| **Rating Scale** | Typically: 5 = “None of the time,” 4 = “A little of the time,” … 1 = “All of the time.” | 5 = “Không bao giờ,” 4 = “Thỉnh thoảng,” … 1 = “Mọi lúc.” | Adapted to same mood/emotions domain style (frequency-based) in Vietnamese. |
