# Supplementary material for: Optimizing health-related quality of life assessments for stroke survivors: a validation study of psychometric properties for the Vietnamese version of stroke impact scale 3.0
Source: Front Public Health. 2025 May 20;13:1570980. doi: 10.3389/fpubh.2025.1570980 (PMC12129923; doi:10.3389/fpubh.2025.1570980)
Supplement: Supplementary file 2 [file Table_2.docx]

**Supplementary 2. Item response analysis of Vietnamese Stroke Impact Scale 3.0**

|  | **Coef.** | **Std. Err.** | **z** | **p-value** | **95% CI** | |
| --- | --- | --- | --- | --- | --- | --- |
| **FACTOR 1: PHYSICAL** |  |  |  |  |  |  |
| **Q1a: Arm that was most affected by your stroke?** | | | | | | |
| Discrimination | 2.03 | 0.23 | 8.79 | 0.000 | 1.57 | 2.48 |
| Difficulty |  |  |  |  |  |  |
| ≥2 |  |  |  |  |  |  |
| ≥3 | -0.31 | 0.10 | -3.16 | 0.002 | -0.51 | -0.12 |
| ≥4 | 0.44 | 0.10 | 4.18 | 0.000 | 0.23 | 0.64 |
| 5 | 1.09 | 0.13 | 7.88 | 0.000 | 0.82 | 1.36 |
| **Q1c: Leg that was most affected by your stroke?** | | | | | | |
| Discrimination | 2.13 | 0.24 | 8.85 | 0.000 | 1.65 | 2.60 |
| Difficulty |  |  |  |  |  |  |
| ≥2 |  |  |  |  |  |  |
| ≥3 | -0.22 | 0.09 | -2.34 | 0.019 | -0.42 | -0.04 |
| ≥4 | 0.56 | 0.11 | 5.21 | 0.000 | 0.35 | 0.77 |
| 5 | 1.17 | 0.14 | 8.25 | 0.000 | 0.88 | 1.44 |
| **Q5d: Clip your toenails?** | | | | | | |
| Discrimination | 3.42 | 0.34 | 9.98 | 0.000 | 2.75 | 4.10 |
| Difficulty |  |  |  |  |  |  |
| ≥2 | -0.89 | 0.10 | -9.09 | 0.000 | -1.08 | -0.70 |
| ≥3 | -0.38 | 0.08 | -4.60 | 0.000 | -0.54 | -0.22 |
| ≥4 | -0.08 | 0.08 | -1.07 | 0.285 | -0.24 | 0.07 |
| 5 | 0.35 | 0.08 | 4.19 | 0.000 | 0.19 | 0.52 |
| **Q5e: Get to the toilet on time?** | | | | | | |
| Discrimination | 3.77 | 0.40 | 9.33 | 0.000 | 2.97 | 4.56 |
| Difficulty |  |  |  |  |  |  |
| ≥2 | -0.29 | 0.08 | -3.64 | 0.000 | -0.45 | -0.13 |
| ≥3 | 0.10 | 0.08 | 1.30 | 0.195 | -0.05 | 0.26 |
| ≥4 | 0.33 | 0.08 | 4.07 | 0.000 | 0.17 | 0.49 |
| 5 | 0.65 | 0.09 | 7.26 | 0.000 | 0.48 | 0.83 |
| **Q5h: Do light household tasks/chores (eg, dust, make a bed, take out garbage, do the dishes)?** | | | | | | |
| Discrimination | 4.17 | 0.43 | 9.77 | 0.000 | 3.34 | 5.01 |
| Difficulty |  |  |  |  |  |  |
| ≥2 | -1.05 | 0.10 | -10.70 | 0.000 | -1.25 | -0.86 |
| ≥3 | -0.63 | 0.08 | -7.43 | 0.000 | -0.79 | -0.46 |
| ≥4 | -0.27 | 0.08 | -3.46 | 0.001 | -0.42 | -0.12 |
| 5 | 0.14 | 0.08 | 1.75 | 0.080 | -0.02 | 0.29 |
| **Q6b: Stay standing without losing your balance?** | | | | | | |
| Discrimination | 6.96 | 0.76 | 9.09 | 0.000 | 5.46 | 8.46 |
| Difficulty |  |  |  |  |  |  |
| ≥2 | -0.80 | 0.08 | -9.70 | 0.000 | -0.96 | -0.64 |
| ≥3 | -0.34 | 0.07 | -4.60 | 0.000 | -0.48 | -0.19 |
| ≥4 | -0.01 | 0.07 | -0.10 | 0.919 | -0.15 | 0.13 |
| 5 | 0.31 | 0.07 | 4.19 | 0.000 | 0.17 | 0.46 |
| **Q6c: Walk without losing your balance?** | | | | | | |
| Discrimination | 8.40 | 1.03 | 8.15 | 0.000 | 6.38 | 10.42 |
| Difficulty |  |  |  |  |  |  |
| ≥2 | -0.85 | 0.08 | -10.40 | 0.000 | -1.02 | -0.69 |
| ≥3 | -0.39 | 0.07 | -5.31 | 0.000 | -0.53 | -0.24 |
| ≥4 | -0.09 | 0.07 | -1.33 | 0.182 | -0.23 | 0.04 |
| 5 | 0.26 | 0.07 | 3.59 | 0.000 | 0.12 | 0.40 |
| **Q6d: Move from a bed to a chair?** | | | | | | |
| Discrimination | 7.76 | 0.92 | 8.40 | 0.000 | 5.95 | 9.58 |
| Difficulty |  |  |  |  |  |  |
| ≥2 | -0.63 | 0.08 | -8.11 | 0.000 | -0.78 | -0.48 |
| ≥3 | -0.22 | 0.07 | -3.10 | 0.002 | -0.36 | -0.08 |
| ≥4 | 0.17 | 0.07 | 2.33 | 0.020 | 0.03 | 0.31 |
| 5 | 0.48 | 0.08 | 6.34 | 0.000 | 0.33 | 0.62 |
| **Q6e: Walk one block?** | | | | | | |
| Discrimination | 9.91 | 1.32 | 7.51 | 0.000 | 7.33 | 12.51 |
| Difficulty |  |  |  |  |  |  |
| ≥2 | -0.66 | 0.08 | -8.61 | 0.000 | -0.81 | -0.51 |
| ≥3 | -0.25 | 0.07 | -3.52 | 0.000 | -0.38 | -0.11 |
| ≥4 | 0.03 | 0.07 | 0.39 | 0.696 | -0.11 | 0.16 |
| 5 | 0.31 | 0.07 | 4.37 | 0.000 | 0.17 | 0.45 |
| **Q6f: Walk fast?** | | | | | | |
| Discrimination | 5.69 | 0.64 | 8.88 | 0.000 | 4.44 | 6.95 |
| Difficulty |  |  |  |  |  |  |
| ≥2 | -1.60 | 0.13 | -12.67 | 0.000 | -1.85 | -1.35 |
| ≥3 | -0.69 | 0.08 | -8.50 | 0.000 | -0.85 | -0.53 |
| ≥4 | -0.35 | 0.08 | -4.69 | 0.000 | -0.50 | -0.21 |
| 5 | 0.07 | 0.07 | 0.94 | 0.346 | -0.08 | 0.21 |
| **Q7a: Carry heavy objects (eg, bag of groceries)?** | | | | | | |
| Discrimination | 3.15 | 0.33 | 9.62 | 0.000 | 2.51 | 3.79 |
| Difficulty |  |  |  |  |  |  |
| ≥2 | -1.72 | 0.15 | -11.12 | 0.000 | -2.03 | -1.42 |
| ≥3 | -0.95 | 0.10 | -9.38 | 0.000 | -1.14 | -0.75 |
| ≥4 | -0.47 | 0.09 | -5.43 | 0.000 | -0.64 | -0.30 |
| 5 | 0.08 | 0.08 | 0.96 | 0.335 | -0.08 | 0.25 |
| **FACTOR 2: COGNITIVE** |  |  |  |  |  |  |
| **Q2a: Remember things that people just told you?** | | | | | | |
| Discrimination | 1.08 | 0.15 | 7.38 | 0.000 | 0.80 | 1.38 |
| Difficulty |  |  |  |  |  |  |
| ≥2 | -1.02 | 0.19 | -5.52 | 0.000 | -1.39 | -0.66 |
| ≥3 | 0.05 | 0.14 | 0.36 | 0.719 | -0.22 | 0.32 |
| ≥4 | 1.45 | 0.23 | 6.27 | 0.000 | 1.00 | 1.90 |
| 5 | 3.19 | 0.44 | 7.32 | 0.000 | 2.34 | 4.05 |
| **Q2b: Remember things that happened yesterday?** | | | | | | |
| Discrimination | 0.93 | 0.14 | 6.82 | 0.000 | 0.66 | 1.20 |
| Difficulty |  |  |  |  |  |  |
| ≥2 | -1.02 | 0.19 | -5.52 | 0.000 | -1.39 | -0.66 |
| ≥3 | 0.05 | 0.14 | 0.36 | 0.719 | -0.22 | 0.32 |
| ≥4 | 1.45 | 0.23 | 6.27 | 0.000 | 1.00 | 1.90 |
| 5 | 3.19 | 0.44 | 7.32 | 0.000 | 2.34 | 4.05 |
| **Q2c: Remember to do things (eg, keep scheduled appointments or take medication)?** | | | | | | |
| Discrimination | 0.95 | 0.14 | 6.91 | 0.000 | 0.69 | 1.23 |
| Difficulty |  |  |  |  |  |  |
| ≥2 | -1.99 | 0.31 | -6.41 | 0.000 | -2.60 | -1.39 |
| ≥3 | -0.53 | 0.16 | -3.21 | 0.001 | -0.85 | -0.20 |
| ≥4 | 0.54 | 0.17 | 3.18 | 0.001 | 0.21 | 0.87 |
| 5 | 2.01 | 0.31 | 6.43 | 0.000 | 1.40 | 2.62 |
| **Q2d: Remember the day of the week?** | | | | | | |
| Discrimination | 1.16 | 0.15 | 7.71 | 0.000 | 0.87 | 1.46 |
| Difficulty |  |  |  |  |  |  |
| ≥2 | -1.33 | 0.20 | -6.55 | 0.000 | -1.73 | -0.93 |
| ≥3 | -0.22 | 0.13 | -1.68 | 0.092 | -0.48 | 0.04 |
| ≥4 | 0.51 | 0.15 | 3.47 | 0.001 | 0.22 | 0.79 |
| 5 | 1.88 | 0.26 | 7.13 | 0.000 | 1.37 | 2.40 |
| **Q2f: Concentrate?** | | | | | | |
| Discrimination | 1.14 | 0.15 | 7.68 | 0.000 | 0.85 | 1.43 |
| Difficulty |  |  |  |  |  |  |
| ≥2 | -1.52 | 0.22 | -6.78 | 0.000 | -1.96 | -1.08 |
| ≥3 | -0.22 | 0.13 | -1.64 | 0.101 | -0.48 | 0.04 |
| ≥4 | 1.00 | 0.18 | 5.56 | 0.000 | 0.65 | 1.36 |
| 5 | 2.43 | 0.32 | 7.51 | 0.000 | 1.80 | 3.06 |
| **Q2g: Think quickly?** | | | | | | |
| Discrimination | 0.99 | 0.14 | 7.03 | 0.000 | 0.71 | 1.27 |
| Difficulty |  |  |  |  |  |  |
| ≥2 | -1.35 | 0.23 | -5.89 | 0.000 | -1.80 | -0.90 |
| ≥3 | -0.31 | 0.15 | -2.05 | 0.041 | -0.60 | -0.01 |
| ≥4 | 0.69 | 0.18 | 3.90 | 0.000 | 0.34 | 1.03 |
| 5 | 2.07 | 0.31 | 6.62 | 0.000 | 1.45 | 2.68 |
| **Q4b: Understand what was being said to you in a conversation?** | | | | | | |
| Discrimination | 1.35 | 0.17 | 8.11 | 0.000 | 1.02 | 1.68 |
| Difficulty |  |  |  |  |  |  |
| ≥2 | -0.13 | 0.12 | -1.06 | 0.288 | -0.37 | 0.11 |
| ≥3 | 1.08 | 0.16 | 6.54 | 0.000 | 0.76 | 1.40 |
| ≥4 | 2.01 | 0.25 | 8.02 | 0.000 | 1.52 | 2.50 |
| 5 | 2.87 | 0.35 | 8.20 | 0.000 | 2.19 | 3.56 |
| **FACTOR 3: SOCIAL PARTICIPATION** | | | | | | |
| **Q8b: Your social activities?** | | | | | | |
| Discrimination | 1.25 | 0.16 | 7.57 | 0.000 | 0.92 | 1.57 |
| Difficulty |  |  |  |  |  |  |
| ≥2 | -1.33 | 0.19 | -7.05 | 0.000 | -1.70 | -0.96 |
| ≥3 | -0.63 | 0.13 | -4.68 | 0.000 | -0.90 | -0.37 |
| ≥4 | 0.07 | 0.13 | 0.55 | 0.581 | -0.18 | 0.32 |
| 5 | 1.03 | 0.18 | 5.56 | 0.000 | 0.66 | 1.39 |
| **Q8c: Quiet recreation (crafts, reading)?** | | | | | | |
| Discrimination | 1.17 | 0.16 | 7.57 | 0.000 | 0.87 | 1.48 |
| Difficulty |  |  |  |  |  |  |
| ≥2 | -1.54 | 0.22 | -7.03 | 0.000 | -1.97 | -1.11 |
| ≥3 | -0.33 | 0.13 | -2.48 | 0.013 | -0.58 | -0.07 |
| ≥4 | 0.47 | 0.15 | 3.16 | 0.002 | 0.18 | 0.76 |
| 5 | 1.72 | 0.25 | 6.79 | 0.000 | 1.22 | 2.22 |
| **Q8d: Active recreation (sports, outings, travel)?** | | | | | | |
| Discrimination | 1.03 | 0.15 | 6.96 | 0.000 | 0.74 | 1.32 |
| Difficulty |  |  |  |  |  |  |
| ≥2 | -2.15 | 0.31 | -6.97 | 0.000 | -2.76 | -1.55 |
| ≥3 | -0.83 | 0.16 | -5.06 | 0.000 | -1.16 | -0.51 |
| ≥4 | 0.09 | 0.15 | 0.60 | 0.548 | -0.20 | 0.38 |
| 5 | 1.31 | 0.24 | 5.55 | 0.000 | 0.85 | 1.78 |
| **Q8f: Your participation in spiritual or religious activities?** | | | | | | |
| Discrimination | 1.27 | 0.16 | 7.84 | 0.000 | 0.95 | 1.59 |
| Difficulty |  |  |  |  |  |  |
| ≥2 | -0.98 | 0.16 | -6.13 | 0.000 | -1.30 | -0.67 |
| ≥3 | -0.19 | 0.12 | -1.50 | 0.134 | -0.43 | 0.06 |
| ≥4 | 0.61 | 0.15 | 4.13 | 0.000 | 0.32 | 0.91 |
| 5 | 1.48 | 0.22 | 6.78 | 0.000 | 1.05 | 1.90 |
| **Q8h: Your ability to control your life as you wish?** | | | | | | |
| Discrimination | 1.10 | 0.15 | 7.15 | 0.000 | 0.80 | 1.40 |
| Difficulty |  |  |  |  |  |  |
| ≥2 | -1.98 | 0.29 | -6.90 | 0.000 | -2.55 | -1.42 |
| ≥3 | -1.02 | 0.18 | -5.74 | 0.000 | -1.37 | -0.67 |
| ≥4 | 0.09 | 0.14 | 0.65 | 0.517 | -0.18 | 0.36 |
| 5 | 1.56 | 0.25 | 6.25 | 0.000 | 1.07 | 2.05 |
| **Q8i: Your ability to help others in need?** | | | | | | |
| Discrimination | 1.13 | 0.16 | 7.22 | 0.000 | 0.83 | 1.44 |
| Difficulty |  |  |  |  |  |  |
| ≥2 | -1.95 | 0.27 | -7.35 | 0.000 | -2.47 | -1.43 |
| ≥3 | -1.02 | 0.16 | -6.17 | 0.000 | -1.34 | -0.69 |
| ≥4 | -0.15 | 0.13 | -1.09 | 0.276 | -0.41 | 0.12 |
| 5 | 1.04 | 0.20 | 5.26 | 0.000 | 0.65 | 1.42 |
| **FACTOR 4: EMOTIONAL** |  |  |  |  |  |  |
| **Q3a: Feel sad?** | | | | | | |
| Discrimination | 1.05 | 0.14 | 7.28 | 0.000 | 0.77 | 1.33 |
| Difficulty |  |  |  |  |  |  |
| ≥2 | -2.10 | 0.30 | -6.94 | 0.000 | -2.70 | -1.51 |
| ≥3 | 0.00 | 0.14 | -0.02 | 0.986 | -0.28 | 0.28 |
| ≥4 | 1.32 | 0.22 | 6.13 | 0.000 | 0.90 | 1.75 |
| 5 | 3.69 | 0.53 | 6.97 | 0.000 | 2.65 | 4.73 |
| **Q3b: Feel that there is nobody you are close to?** | | | | | | |
| Discrimination | 1.08 | 0.15 | 7.39 | 0.000 | 0.80 | 1.37 |
| Difficulty |  |  |  |  |  |  |
| ≥2 | -0.85 | 0.17 | -4.94 | 0.000 | -1.19 | -0.51 |
| ≥3 | 0.47 | 0.15 | 3.11 | 0.002 | 0.17 | 0.76 |
| ≥4 | 1.68 | 0.25 | 6.67 | 0.000 | 1.18 | 2.17 |
| 5 | 3.79 | 0.56 | 6.76 | 0.000 | 2.69 | 4.89 |
| **Q3c: Feel that you are a burden to others?** | | | | | | |
| Discrimination | 1.15 | 0.15 | 7.77 | 0.000 | 0.86 | 1.44 |
| Difficulty |  |  |  |  |  |  |
| ≥2 | -0.88 | 0.17 | -5.32 | 0.000 | -1.21 | -0.56 |
| ≥3 | 0.40 | 0.14 | 2.84 | 0.005 | 0.12 | 0.67 |
| ≥4 | 1.52 | 0.22 | 6.82 | 0.000 | 1.09 | 1.96 |
| 5 | 3.86 | 0.54 | 7.20 | 0.000 | 2.81 | 4.91 |
| **Q3d: Feel that you have nothing to look forward to?** | | | | | | |
| Discrimination | 1.08 | 0.15 | 7.42 | 0.000 | 0.80 | 1.37 |
| Difficulty |  |  |  |  |  |  |
| ≥2 | -0.45 | 0.15 | -3.00 | 0.003 | -0.75 | -0.16 |
| ≥3 | 0.85 | 0.17 | 5.05 | 0.000 | 0.52 | 1.18 |
| ≥4 | 1.99 | 0.28 | 7.16 | 0.000 | 1.44 | 2.53 |
| 5 | 4.23 | 0.62 | 6.78 | 0.000 | 3.01 | 5.46 |
| **Q3e: Blame yourself for mistakes?** | | | | | | |
| Discrimination | 0.92 | 0.14 | 6.46 | 0.000 | 0.64 | 1.19 |
| Difficulty |  |  |  |  |  |  |
| ≥2 | -0.09 | 0.16 | -0.60 | 0.550 | -0.41 | 0.22 |
| ≥3 | 1.12 | 0.22 | 5.03 | 0.000 | 0.68 | 1.56 |
| ≥4 | 2.65 | 0.42 | 6.32 | 0.000 | 1.83 | 3.47 |
| 5 | 5.05 | 0.86 | 5.86 | 0.000 | 3.36 | 6.74 |

**Caption**: **Supplementary 2** illustrates the item response analysis results for the Vietnamese Stroke Impact Scale 3.0. Discrimination parameters and threshold (difficulty) estimates are provided for each item, along with their standard errors, z-values, p-values, and 95% confidence intervals. The analysis is organized by factor (Physical, Cognitive, Social Participation, and Emotional), with discrimination parameters indicating how well an item differentiates between respondents at different levels of the latent trait, and difficulty parameters reflecting the thresholds for transitioning between response categories.
